# The association between maternal FT3/FT4 ratio in early pregnancy and adverse neonatal outcomes: a retrospective cohort study

**DOI:** 10.3389/fendo.2026.1802981

**Published:** 2026-05-12

**Authors:** Xiaoyu Chen, Fuyu Yang, Fanlong Meng, Lixin Li, Zhongyang Han, Jiayue Wang, Qingliang Shao, Shuang Li, Wei Sun

**Affiliations:** 1 The Fourth Affiliated Hospital of Harbin Medical University, Harbin, Heilongjiang, China; 2 The Affiliated Stomatology Hospital of Jiamusi University, Jiamusi, Heilongjiang, China; 3 The First Affiliated Hospital of Huzhou Normal University, Huzhou First People’s Hospital, Huzhou, Zhejiang, China

**Keywords:** deiodinase activity, FT3/FT4 ratio, maternal thyroid function, neonatal adverse outcomes, pregnancy, thyroid homeostasis

## Abstract

**Introduction:**

Maternal thyroid hormone homeostasis during early gestation is fundamental for fetal growth and development, yet the specific clinical implications of the ratio between free triiodothyronine and free thyroxine, a marker of peripheral deiodinase activity and metabolic status, remain insufficiently explored regarding newborn health.

**Methods:**

This retrospective cohort study investigated the association between the maternal free triiodothyronine to free thyroxine ratio measured during the first trimester and the risk of neonatal adverse outcomes. We analyzed data from 797 eligible mother–infant pairs, excluding those with pre-existing thyroid or immune disorders to isolate physiological variations. The primary endpoint was a composite of neonatal adverse outcomes, including respiratory, cardiovascular, and infectious complications. Multivariable logistic regression and restricted cubic spline modeling were employed to evaluate linear and non-linear associations.

**Results:**

The results demonstrated that mothers in the highest quartile of the thyroid hormone ratio exhibited a significantly increased risk of adverse neonatal outcomes compared to those in the lowest quartile, an association that remained robust after adjusting for potential confounders such as maternal age, body mass index, and gestational age. The relationship followed a non-linear J-shaped pattern, with risks escalating sharply beyond a specific threshold. Specifically, a higher ratio was strongly associated with neonatal anemia, patent ductus arteriosus, jaundice, and myocardial injury. Subgroup analyses revealed that this risk was particularly pronounced in nulliparous women, mothers with a normal body mass index, and female neonates.

**Discussion:**

These findings suggest that an elevated free triiodothyronine to free thyroxine ratio in early pregnancy serves as a sensitive indicator of neonatal risk, potentially reflecting maladaptive maternal metabolic demands that impact fetal health independent of traditional obstetric risk factors.

## 1 Introduction

Thyroid hormones are pivotal regulators of physiological homeostasis, governing metabolism, cellular growth, and fetal neurodevelopment (1). During pregnancy, the maternal thyroid gland undergoes significant adaptive changes to meet the heightened metabolic demands of the mother and the developing fetus (2). While thyroxine (T4) is the primary pro-hormone secreted by the thyroid, it is free triiodothyronine (FT3) that serves as the biologically active form, exerting potent effects on cellular energy expenditure and metabolic regulation. Conventionally, the assessment of thyroid function in pregnancy has relied heavily on thyroid-stimulating hormone (TSH) and free thyroxine (FT4). However, relying solely on these markers may overlook subtle alterations in thyroid homeostasis, particularly in euthyroid women whose levels fall within population-based reference ranges (3, 4).

Emerging evidence suggests that the conversion efficiency of T4 to FT3, often reflected by the FT3/FT4 ratio, serves as a critical indicator of peripheral thyroid sensitivity and deiodinase activity (5–8). This conversion is highly sensitive to metabolic status, nutritional factors, and oxidative stress (9–11). In the context of pregnancy, an elevated conversion rate or disproportionate FT3 levels relative to FT4 may reflect a maternal compensatory response to metabolic stress or systemic inflammation. Despite the physiological importance of this mechanism, the clinical implications of the FT3/FT4 ratio in early pregnancy remain under-investigated compared to traditional thyroid markers (12, 13).Unlike TSH, which primarily reflects the central hypothalamic-pituitary-thyroid feedback loop, the FT3/FT4 ratio serves as a surrogate marker for peripheral deiodinase activity and tissue-level thyroid sensitivity. In euthyroid pregnancies, an elevated ratio may may reflect a maternal compensatory response to subclinical metabolic stress or systemic inflammation, which conventional thyroid markers often fail to detect. Understanding this ‘peripheral thyroid phenotype’ is crucial for identifying high-risk pregnancies that would otherwise be classified as normal under current screening guidelines.

Neonatal adverse outcomes (NAOs), including low birth weight, small for gestational age (SGA), and preterm birth, represent significant public health challenges associated with long-term morbidity. The etiology of NAOs is multifactorial, involving placental dysfunction, impaired angiogenesis, and maternal maladaptation to pregnancy. Given that thyroid hormones directly influence placental development and maternal cardiovascular adaptation, it is hypothesized that dysregulated thyroid hormone conversion—manifesting as an altered FT3/FT4 ratio—could contribute to the pathogenesis of these adverse outcomes (1, 14). Specifically, excessive FT3 activity or an imbalance in the FT3/FT4 ratio may exacerbate oxidative stress and metabolic dysregulation, thereby impairing the intrauterine environment.

Although previous studies have established the risks of overt hypothyroidism and hyperthyroidism, the prognostic value of the FT3/FT4 ratio in euthyroid pregnant women is not well established (6, 13). Identifying early, sensitive biomarkers is crucial for risk stratification and the improvement of perinatal care. Therefore, this study aims to retrospectively investigate the association between the maternal serum FT3/FT4 ratio in early pregnancy and the risk of neonatal adverse outcomes. By analyzing a cohort of 797 pregnancies, we seek to determine whether this ratio can serve as a novel predictor for NAOs, thereby providing new insights into the role of thyroid homeostasis in fetal health.

## 2 Materials and methods

### 2.1 Study design and participants

We conducted a retrospective cohort study involving 797 mother–infant pairs managed at the Fourth Hospital of Harbin Medical University between 2016 and 2024. The study population consisted of women who underwent routine antenatal care and delivery at the study center.

Inclusion Criteria: Participants were included if they met the following criteria: (1) underwent quantitative thyroid function testing during the first trimester (8–13 gestational weeks); (2) had a singleton pregnancy; and (3) had complete medical records regarding maternal demographics and neonatal outcomes.

Exclusion Criteria: To isolate the effect of physiological thyroid hormone variations, we applied strict exclusion criteria. Women were excluded if they had: (1) a history of thyroid disease or thyroid surgery; (2) preexisting autoimmune or systemic immune-mediated disorders; (3) significant pre-pregnancy comorbidities (e.g., chronic hypertension, diabetes mellitus, renal or liver disease); (4) multiple gestations or conception via assisted reproductive technology; (5) exposure to medications affecting thyroid function or immune status (e.g., levothyroxine, antithyroid drugs, glucocorticoids); or (6) fetal congenital anomalies. A detailed list of exclusion criteria is provided in **Supplementary Table 1**.

This study was conducted and reported in accordance with the Strengthening the Reporting of Observational Studies in Epidemiology (STROBE) guidelines for cohort studies.

### 2.2 Ethical approval

The study protocol was reviewed and approved by the Ethics Committee of the Fourth Hospital of Harbin Medical University (Approval No. 2022-WZYSLLSC-14). The requirement for informed consent was waived due to the retrospective nature of the study and the use of anonymized data, in accordance with the Declaration of Helsinki and institutional regulations.

### 2.3 Laboratory measurements and clinical data collection

Maternal venous blood samples were collected during the first trimester (8–13 weeks). Serum levels of free triiodothyronine (FT3), free thyroxine (FT4), and thyroid-stimulating hormone (TSH) were quantified using chemiluminescent immunoassays at the Department of Laboratory Medicine, adhering to rigorous quality control standards. The FT3/FT4 ratio was calculated as the serum FT3 concentration divided by the serum FT4 concentration. Maternal demographic and clinical data—including age, pre-pregnancy body mass index (BMI), parity, and pregnancy-related complications—were extracted from electronic medical records (EMR). Neonatal anthropometric data (sex, birth weight, birth length) were recorded postpartum.

### 2.4 Covariates

Covariates were selected *a priori* based on clinical relevance and established associations with thyroid physiology and pregnancy outcomes. The following variables were included in adjusted models: maternal age, pre-pregnancy BMI, gravidity, parity, gestational hypertension, gestational diabetes, renal function markers (serum creatinine, uric acid), hematologic markers (white blood cell count, platelet count), TSH, and thyroid autoantibodies (thyroid peroxidase antibody [TPOAb], thyroglobulin antibody [TgAb]).

### 2.5 Definition of neonatal adverse outcomes

The primary endpoint was a composite of Neonatal Adverse Outcomes (NAOs). NAOs were ascertained from the neonatal EMR system based on clinician-assigned diagnoses. The composite endpoint was defined as the occurrence of at least one adverse event during the birth hospitalization, including but not limited to: hypoxic–ischemic encephalopathy (HIE), respiratory distress syndrome (RDS), bronchopulmonary dysplasia (BPD), myocardial injury, neonatal sepsis, and necrotizing enterocolitis (NEC). A comprehensive list of the specific diagnoses included in the composite endpoint, along with their frequencies in the cohort, is detailed in **Supplementary Table 2**. Diagnoses of NOAs were made by attending neonatologists based on standard clinical guidelines. As this was a retrospective study, the clinical team was blinded to the maternal FT3/FT4 ratio groupings and the specific research hypothesis at the time of diagnosis, thereby minimizing detection bias.

### 2.6 Statistical analysis

Statistical analyses were performed using R software (version 4.2.0). Continuous variables were presented as mean ± standard deviation (SD) or median (interquartile range, IQR) based on normality, while categorical variables were expressed as frequencies (percentages). Differences across quartiles of the FT3/FT4 ratio were assessed using ANOVA, the Kruskal–Wallis test, or the Chi-square test, as appropriate.To assess the potential impact of unmeasured confounding, we calculated the E-value for the fully adjusted model (Model 3). The E-value quantifies the minimum strength of association that an unmeasured confounder would need to have with both the exposure and the outcome to explain away the observed association.

Multivariable logistic regression was employed to estimate odds ratios (ORs) and 95% confidence intervals (CIs) for the association between the FT3/FT4 ratio (categorized into quartiles, with Q1 as the reference) and NAOs. Three models were constructed: **Model 1**: Adjusted for laboratory covariates (WBC, platelets, creatinine, uric acid). **Model 2** (Primary Model): Further adjusted for maternal age, pre-pregnancy BMI, gravidity, parity, gestational hypertension, gestational diabetes, TSH, TPOAb, and TgAb. **Model 3** (Fully adjusted Analysis): Additionally adjusted for potential mediators, including neonatal sex, gestational age at delivery, and birth weight.

To evaluate potential non-linear relationships, restricted cubic splines (RCS) with four knots were fitted for the continuous FT3/FT4 ratio. Subgroup analyses were conducted to assess effect modification by maternal age, BMI, and comorbidities, with interactions tested using the Wald chi-square test. Statistical significance was defined as a two-sided p-value < 0.05.

## 3 Results

### 3.1 Baseline characteristics of the study population

A total of 797 eligible mother–infant pairs were included in the final analysis. Based on the presence of the composite endpoint, the cohort was stratified into two groups: those without NAOs (n = 419, 52.6%) and those with NAOs (n = 378, 47.4%). A detailed comparison of demographic, clinical, and laboratory characteristics between these groups is presented in **Supplementary Table 3**.

Maternal demographic and anthropometric analyses revealed significant differences in body composition between the groups. Mothers in the NAO group exhibited a significantly higher pre-pregnancy weight (p = 0.036) and body mass index (BMI) compared to those in the non-NAO group (mean 28.36 ± 4.32 kg/m² vs. 27.75 ± 4.05 kg/m²; p = 0.040). While maternal age and height were comparable, the NAO group demonstrated a higher burden of clinical risk factors. Specifically, mothers with affected infants had significantly higher diastolic blood pressure (p = 0.013) and a marginally higher systolic blood pressure (p = 0.060). Furthermore, the prevalence of maternal pregnancy complications was substantially elevated in the NAO group (76.7%) compared to the non-NAO group (38.4%; p < 0.001). Obstetric history also differed slightly, with the NAO group reporting higher gravidity (p = 0.025), although parity remained similar between groups.

Regarding laboratory parameters, the NAO group displayed a distinct inflammatory and thyroid function profile. White blood cell counts were significantly elevated in mothers whose infants developed adverse outcomes (*p* < 0.001). Crucially, significant disparities were observed in all measured thyroid function markers. Mothers in the NAO group had significantly higher median levels of free triiodothyronine (FT3) and free thyroxine (FT4) compared to controls (both *p* < 0.001). Consequently, the FT3/FT4 ratio was significantly higher in the NAO group (median 0.31, IQR [0.28–0.34]) compared to the non-NAO group (median 0.29, IQR [0.27–0.32]; *p* < 0.001). Consistent with the negative feedback loop of the hypothalamic-pituitary-thyroid axis, TSH levels were significantly lower in the NAO group (*p* = 0.017). Moreover, markers of thyroid autoimmunity were markedly elevated in the NAO group; median titers for both thyroglobulin antibody (TgAb) and thyroid peroxidase antibody (TPOAb) were significantly higher compared to the non-NAO group (*p* < 0.001 for both), suggesting a potential link between maternal thyroid autoimmunity and adverse neonatal outcomes.

Neonatal characteristics at delivery confirmed the severity of the composite outcome. Infants in the NAO group had significantly lower birth weights and gestational ages compared to those in the non-NAO group (*p* < 0.001 for both). Notably, 33.2% of infants in the NAO group had a birth weight between 1500–2499 g, and 32.8% were born between 34–36 gestational weeks, whereas the vast majority of infants in the non-NAO group were born at term with normal birth weights. Neonatal sex distribution did not differ significantly between the two groups (*p* = 0.090).

### 3.2 Baseline characteristics stratified by FT3/FT4 ratio quartiles

To further elucidate the clinical relevance of thyroid hormone balance, participants were stratified into four groups based on quartiles of the FT3/FT4 ratio (Q1 to Q4). As detailed in **Table 1**, demographic and anthropometric analyses revealed significant heterogeneity across these groups. While maternal age varied significantly among the quartiles (*p* = 0.013), with the lowest quartile (Q1) exhibiting the highest mean age, the most pronounced differences were observed in body composition. Both maternal weight and Body Mass Index (BMI) demonstrated a robust, positive linear trend across the quartiles (*p* < 0.001 for both). Participants in the highest quartile (Q4) had a significantly higher mean BMI (29.05 ± 4.34 kg/m²) compared to those in the lowest quartile (26.93 ± 3.41 kg/m²). Notably, our baseline analysis revealed substantial differences across FT3/FT4 ratio quartiles: women in the lowest quartile (Q1) were significantly older, had lower body weight, and lower BMI compared to those in higher quartiles. These differences suggest that the FT3/FT4 ratio is closely linked to maternal metabolic status and body composition. Lower BMI and body weight in Q1 may reflect reduced substrate availability for thyroid hormone production and altered peripheral deiodinase activity, which is known to be influenced by nutritional status and adiposity.

**Table 1:** Baseline characteristics of participants according to FT3/FT4 quartile groups.

Variable	Q1 (n = 200)	Q2 (n = 199)	Q3 (n = 199)	Q4 (n = 199)	*p*-value
Maternal age (years)^a^	31.24 (3.81)	30.23 (3.56)	30.11 (3.98)	30.51 (3.75)	0.013
Height (cm)^a^	163.68 (4.93)	163.16 (6.02)	162.98 (5.23)	163.09 (5.11)	0.572
Weight (kg)^a^	72.25 (10.28)	74.79 (11.76)	74.52 (11.69)	77.30 (11.61)	<0.001
Body Mass Index (BMI)^a^	26.93 (3.41)	28.15 (4.70)	28.04 (3.94)	29.05 (4.34)	<0.001
Systolic blood pressure (mmHg)^b^	114.00 [110.00, 120.00]	110.00 [110.00, 119.00]	110.50 [110.00, 120.00]	117.00 [110.00, 120.00]	0.140
Diastolic blood pressure (mmHg)^b^	72.50 [70.00, 80.00]	70.00 [70.00, 80.00]	73.50 [70.00, 80.00]	78.50 [70.00, 80.00]	0.032
Gravidity (G)^a^	1.61 (0.84)	1.58 (0.91)	1.68 (0.85)	1.83 (1.04)	0.036
Parity (P)^a^	1.14 (0.36)	1.13 (0.37)	1.12 (0.34)	1.16 (0.43)	0.814
White blood cell count (×10⁹/L)^a^	10.25 (3.22)	10.09 (3.06)	10.42 (2.72)	10.49 (3.26)	0.577
Platelet count (×10⁹/L)^a^	206.94 (59.52)	205.51 (52.56)	201.70 (57.30)	199.70 (53.05)	0.542
Serum creatinine (µmol/L)^b^	47.40 [42.08, 54.00]	45.60 [40.40, 52.35]	45.75 [40.58, 49.92]	46.10 [41.00, 53.00]	0.123
Uric acid (µmol/L)^b^	281.50 [233.98, 330.08]	278.00 [235.12, 339.05]	276.00 [230.30, 329.25]	286.00 [242.60, 342.95]	0.427
Free triiodothyronine (FT3, pmol/L)^b^	4.06 [3.71, 4.46]	4.37 [4.03, 4.70]	4.47 [4.14, 4.78]	4.82 [4.40, 5.18]	<0.001
Free thyroxine (FT4, pmol/L)^b^	16.55 [15.01, 17.98]	15.31 [14.25, 16.51]	14.29 [13.29, 15.30]	13.61 [12.12, 14.54]	<0.001
FT3/FT4 ratio^b^	0.25 [0.24, 0.26]	0.29 [0.28, 0.29]	0.31 [0.30, 0.32]	0.35 [0.34, 0.37]	<0.001
Thyroid-stimulating hormone (TSH, mIU/L)^b^	1.64 [0.98, 2.33]	1.51 [1.02, 2.30]	1.53 [1.04, 2.16]	1.50 [1.03, 2.18]	0.892
Thyroglobulin antibody (TgAb, IU/mL)^b^	3.55 [0.90, 16.51]	10.61 [0.90, 25.10]	10.61 [0.90, 21.73]	16.63 [5.74, 28.85]	<0.001
Anti-thyroid peroxidase antibody (TPOAb, IU/mL)^b^	5.86 [0.25, 28.00]	22.37 [0.28, 32.79]	16.98 [0.33, 30.98]	28.00 [5.24, 35.95]	<0.001
Low birth weight (n, %)	163 (82.7)	154 (78.2)	160 (82.1)	153 (77.7)	0.471
Premature birth (n, %)	153 (76.9)	150 (76.5)	141 (71.9)	135 (73.0)	0.588
Neonatal sex (Male, n, %)	102 (51.0)	99 (49.7)	103 (51.8)	102 (51.3)	0.982
Maternal outcome (With complications, n, %)	121 (60.5)	101 (50.8)	110 (55.3)	119 (59.8)	0.173

Variables are presented as mean ± SD “a” or median [IQR] “b”. Statistical significance was set at p < 0.05.

Regarding clinical and obstetric history, significant differences were noted in hemodynamic and reproductive parameters. Mothers in the higher quartiles presented with significantly elevated diastolic blood pressure (*p* = 0.032) and a higher frequency of gravidity (*p* = 0.036) compared to those in the lower quartiles. However, other baseline clinical markers, including systolic blood pressure, parity, white blood cell count, platelet count, serum creatinine, and uric acid levels, did not differ significantly among the groups (*p* > 0.05).

Thyroid function profiles exhibited distinct patterns consistent with the stratification method. From Q1 to Q4, median FT3 levels increased significantly, while median FT4 levels showed a corresponding significant decrease (*p* < 0.001 for both). Interestingly, despite these significant shifts in peripheral thyroid hormone concentrations, central thyroid regulation appeared stable, as indicated by comparable TSH levels across all quartiles (*p* = 0.892). Notably, a strong association was observed between the FT3/FT4 ratio and markers of thyroid autoimmunity. Titers for both thyroglobulin antibody (TgAb) and anti-thyroid peroxidase antibody (TPOAb) were significantly elevated in the higher quartiles (*p* < 0.001), suggesting a potential link between a higher FT3/FT4 ratio and increased thyroid autoimmune activity.

Despite the significant metabolic and hormonal differences observed across the quartiles, the incidence of adverse pregnancy outcomes did not vary significantly in this unadjusted comparison. The rates of low birth weight, premature birth, and maternal complications were comparable across the four groups (*p* > 0.05), and neonatal sex distribution remained uniform (*p* = 0.982).

### 3.3 Association between maternal FT3/FT4 ratio and neonatal adverse outcomes

The association between maternal FT3/FT4 ratio quartiles and neonatal adverse outcomes was analyzed using multivariable logistic regression models, as presented in **Table 2**. In the crude analysis, participants in the highest quartile (Q4) of the FT3/FT4 ratio demonstrated significantly higher odds of neonatal adverse outcomes compared to those in the lowest quartile (Q1) (OR = 2.232, 95% CI: 1.495–3.331, *p* < 0.001).

**Table 2:** Association between maternal FT3/FT4 ratio quartiles and neonatal adverse outcomes.

FT3/FT4 Quartile	Crude Model			Adjusted Model 1			Adjusted Model 2			Adjusted Model 3		
	OR	P	95% CI	OR	P	95% CI	OR	P	95% CI	OR	P	95% CI
Q1	1 reference			1 reference			1 reference			1 reference		
Q2	1.118	0.583	(0.751, 1.665)	1.206	0.376	(0.797, 1.824)	1.195	0.412	(0.781, 1.830)	1.262	0.392	(0.741, 2.149)
Q3	1.238	0.293	(0.832, 1.841)	1.219	0.353	(0.803, 1.850)	1.196	0.412	(0.780, 1.832)	1.101	0.737	(0.629, 1.928)
Q4	2.232	0.000	(1.495, 3.331)	2.355	0.000	(1.544, 3.591)	2.195	0.000	(1.422, 3.388)	2.690	0.001	(1.540, 4.696)

OR, odds ratio; P, p-value; CI, confidence interval. Q1 is used as the reference group.

Adjusted Model 1: Adjusted for white blood cell count, platelet count, serum creatinine, and uric acid.

Adjusted Model 2: Adjusted for white blood cell count, platelet count, serum creatinine, uric acid, maternal age at delivery, maternal BMI, gravidity, and parity.

Adjusted Model 3: Adjusted for white blood cell count, platelet count, serum creatinine, uric acid, maternal age at delivery, maternal BMI, gravidity, parity, neonatal sex, neonatal birth weight, and gestational age at delivery.

Statistical significance was set at p < 0.05.

This association remained robust after adjusting for potential confounders. In Model 1, adjusting for maternal hematological and renal function parameters, the odds ratio for Q4 remained significant (OR = 2.355, 95% CI: 1.544–3.591, *p* < 0.001). Similarly, in Model 2, which further adjusted for maternal demographic and obstetric characteristics (age, BMI, gravidity, and parity), the elevated odds for the highest quartile persisted (OR = 2.195, 95% CI: 1.422–3.388, *p* < 0.001).

In the fully adjusted Model 3, after controlling for all covariates including neonatal sex, birth weight, and gestational age at delivery, the association was further strengthened. Mothers in Q4 had a 2.69-fold increase in the odds of neonatal adverse outcomes compared to the reference group (OR = 2.690, 95% CI: 1.540–4.696, *p* = 0.001). Conversely, no statistically significant associations were observed for the second (Q2) and third (Q3) quartiles across all models (*p* > 0.05), suggesting that the increased risk may be specific to the highest range of the FT3/FT4 ratio.

To evaluate the total effect of maternal thyroid function on neonatal outcomes and assess the robustness of our findings, we performed a sensitivity analysis excluding neonatal birth weight and gestational age—potential mediators of the association—from the multivariable model. As presented in **Supplementary Table 4**, the association remained robust. Compared with the lowest quartile (Q1), neonates in the highest quartile (Q4) of the maternal FT3/FT4 ratio exhibited a significantly increased risk of the outcome (OR = 2.218, 95% CI: 1.431–3.438, *P* < 0.0001). This suggests that the adverse impact of an elevated FT3/FT4 ratio persists and is statistically significant, representing a strong total effect independent of adjustment for fetal growth parameters.

To further explore the specific types of adverse outcomes driven by thyroid function, we performed separate logistic regression analyses for individual NAOs, as detailed in **Table 3**. In the fully adjusted models (Model 3), the highest quartile (Q4) of the maternal FT3/FT4 ratio remained significantly associated with four specific outcomes compared to the lowest quartile (Q1). The strongest association was observed for **Anemia of the Newborn**, where mothers in Q4 had a more than 4-fold increase in odds compared to those in Q1 (adjusted OR = 4.127, 95% CI: 1.614–10.553, *p* = 0.003). Additionally, significant elevated odds in the Q4 group were found for **Patent Ductus Arteriosus** (adjusted OR = 2.408, 95% CI: 1.134–5.114, *p* = 0.022), **Neonatal Jaundice** (adjusted OR = 1.924, 95% CI: 1.179–3.140, *p* = 0.009), and **Myocardial Injury** (adjusted OR = 1.780, 95% CI: 1.072–2.955, *p* = 0.026). Notably, the association between the FT3/FT4 ratio and NAOs was further strengthened in Model 3 after adjusting for gestational age and birth weight (OR = 2.690, 95% CI: 1.540–4.696; p = 0.001). This increased effect size suggests a ‘suppressor effect, ‘ indicating that maternal thyroid hormone conversion may affect neonatal health through pathological pathways independent of fetal growth restriction or preterm delivery.

**Table 3:** Association between maternal FT3/FT4 ratio quartiles and specific neonatal adverse outcomes.

FT3/FT4 Quartile	Crude Model			Adjusted Model 1			Adjusted Model 2			Adjusted Model 3		
	OR	P	95% CI	OR	P	95% CI	OR	P	95% CI	OR	P	95% CI
Patent Ductus Arteriosus												
Q2	1.402	0.358	(0.682, 2.883)	1.564	0.236	(0.747, 3.274)	1.497	0.300	(0.699, 3.207)	1.455	0.336	(0.678, 3.125)
Q3	1.484	0.278	(0.727,3.030)	1.656	0.176	(0.798, 3.436)	1.860	0.098	(0.892, 3.876)	1.628	0.212	(0.758, 3.497)
Q4	2.086	0.034	(1.058, 4.110)	2.306	0.018	(1.158, 4.594)	2.767	0.006	(1.344, 5.700)	2.408	0.022	(1.134, 5.114)
Myocardial Injury												
Q2	1.152	0.544	(0.731, 1.815)	1.252	0.348	(0.783, 2.001)	1.316	0.259	(0.817, 2.121)	1.336	0.242	(0.822, 2.170)
Q3	1.372	0.166	(0.878,2.145)	1.382	0.171	(0.870, 2.194)	1.412	0.151	(0.882, 2.259)	1.315	0.275	(0.805,2.148)
Q4	1.969	0.002	(1.274, 3.042)	2.105	0.001	(1.339, 3.310)	2.033	0.003	(1.273, 3.248)	1.780	0.026	(1.072,2.955)
Neonatal Jaundice												
Q2	1.008	0.972	(0.665, 1.527)	1.069	0.758	(0.699, 1.636)	1.081	0.727	(0.698, 1.676)	0.947	0.825	(0.582, 1.539)
Q3	1.175	0.442	(0.779,1.773)	1.161	0.491	(0.759, 1.775)	1.200	0.410	(0.778, 1.849)	1.013	0.956	(0.634,1.619)
Q4	1.888	0.002	(1.260, 2.829)	1.957	0.002	(1.286, 2.977)	2.002	0.002	(1.297, 3.089)	1.924	0.009	(1.179,3.140)
Anemia of the Newborn												
Q2	0.495	0.325	(0.122, 2.009)	0.504	0.338	(0.124, 2.046)	0.480	0.301	(0.119, 1.927)	0.504	0.337	(0.125, 2.040)
Q3	1.711	0.308	(0.609,4.804)	1.712	0.311	(0.605, 4.847)	1.607	0.381	(0.557, 4.638)	1.819	0.271	(0.627,5.277)
Q4	3.815	0.005	(1.504, 9.672)	3.797	0.005	(1.489, 9.682)	3.650	0.009	(1.379, 9.663)	4.127	0.003	(1.614,10.553)

OR, odds ratio; P, p-value; CI, confidence interval. Q1 is used as the reference group..

Adjusted Model 1: Adjusted for white blood cell count, platelet count, serum creatinine, and uric acid.

Adjusted Model 2: Adjusted for white blood cell count, platelet count, serum creatinine, uric acid, maternal age at delivery, ma-ternal BMI, gravidity, and parity.

Adjusted Model 3: Adjusted for white blood cell count, platelet count, serum creatinine, uric acid, maternal age at delivery, ma-ternal BMI, gravidity, parity, neonatal sex, neonatal birth weight, and gestational age at delivery. Q1 as reference. Statistical significance was set at p < 0.05.

### 3.4 Dose-response relationship between maternal FT3/FT4 ratio and neonatal outcomes

**Figure 1:**
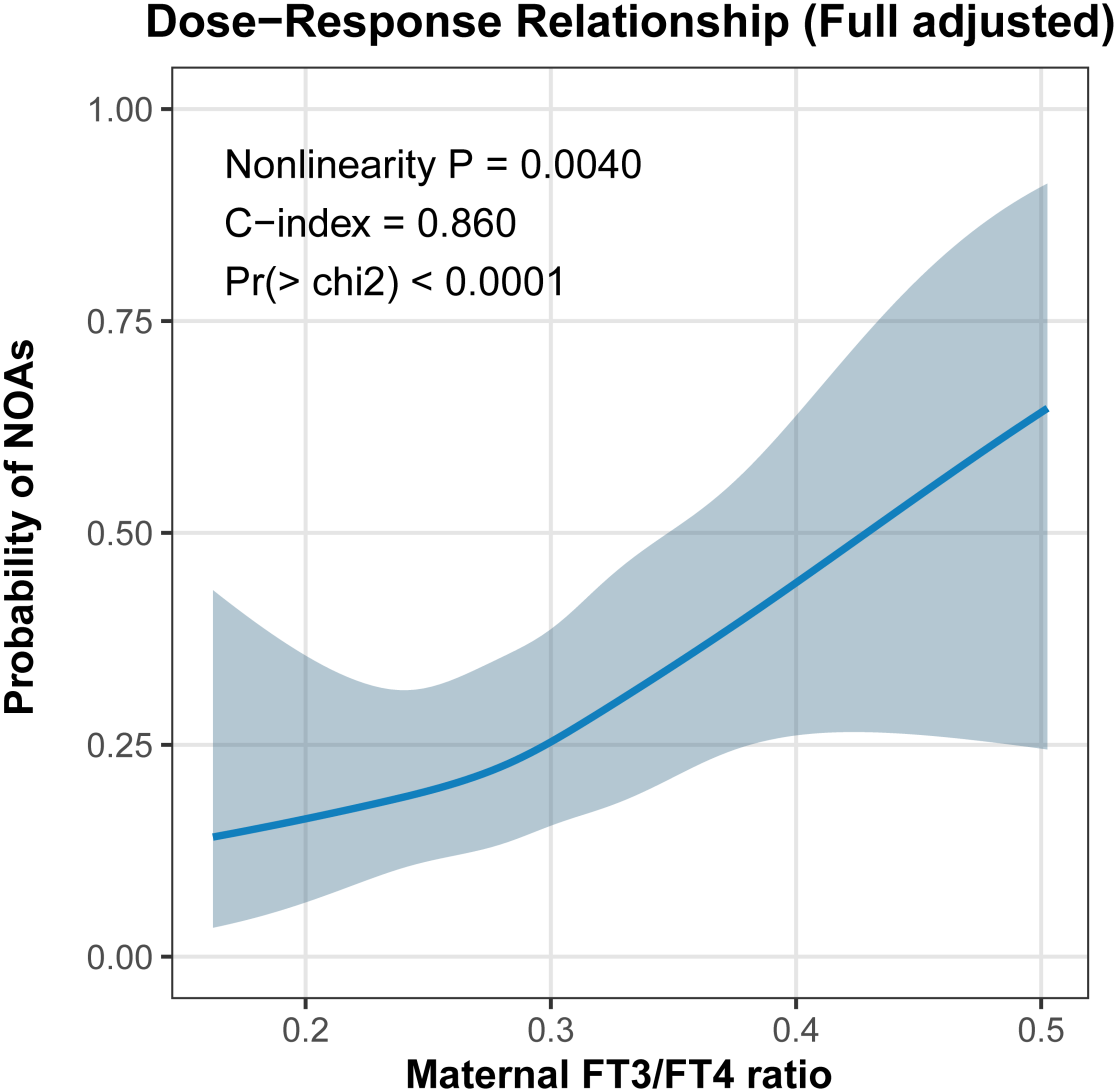
Restricted cubic spline analysis of the association between maternal FT3/FT4 ratio and neonatal adverse outcomes.

We further employed restricted cubic spline (RCS) regression to visualize the continuous relationships between the maternal FT3/FT4 ratio and the risk of neonatal adverse outcomes, adjusting for all potential confounders. As shown in **Figure 1**, there was a significant non-linear positive association between the FT3/FT4 ratio and the composite outcome of NAOs (*P* for non-linearity = 0.0040). The dose-response curve exhibited a “J-shaped” pattern: the probability of NAOs remained relatively flat and low when the ratio was below approximately 0.25, but surged rapidly as the ratio increased beyond this threshold. The shaded area representing the 95% confidence interval widened at the higher end of the ratio distribution, reflecting smaller sample sizes in the extreme ranges, yet the upward trend remained robust. The multivariable model demonstrated excellent predictive performance for the composite outcome, achieving a C-index of 0.860.

The analysis of individual outcomes, presented in **Supplementary Figure 1**, revealed heterogeneous patterns of association. As shown in **Supplementary Figure 1A and 1B**, the associations for patent ductus arteriosus (PDA) and myocardial injury did not exhibit significant non-linearity (P for non-linearity = 0.068 and 0.163, respectively). In contrast, a distinct non-linear relationship was observed for neonatal jaundice (*P* for non-linearity = 0.011) in **Supplementary Figure 1C**, mirroring the trend seen in the composite outcome. The risk of jaundice was relatively stable at lower FT3/FT4 levels but escalated sharply once the ratio exceeded 0.30, with the model showing strong discrimination (C-index = 0.818). Finally, regarding preterm anemia (**Supplementary Figure 1D**), while the visual inspection of the curve suggested a threshold effect where risk increased dramatically only at the highest quartile of the FT3/FT4 ratio, the statistical test for non-linearity was not significant (*P* = 0.643). This indicates that, statistically, the relationship is best described as linear within the current sample distribution. Notably, the fully adjusted model for preterm anemia yielded an exceptionally high C-index of 0.948. This superior discrimination likely reflects the strong influence of other adjusted covariates—such as gestational age and birth weight—in predicting anemia, in conjunction with the contribution of thyroid function markers. Collectively, these RCS analyses reinforce the finding that an elevated FT3/FT4 ratio is a consistent risk marker across multiple neonatal domains, with particular potency at the upper extremes of the distribution.

### 3.5 Subgroup analyses of the association between maternal FT3/FT4 ratio and neonatal outcomes

**Figure 2:**
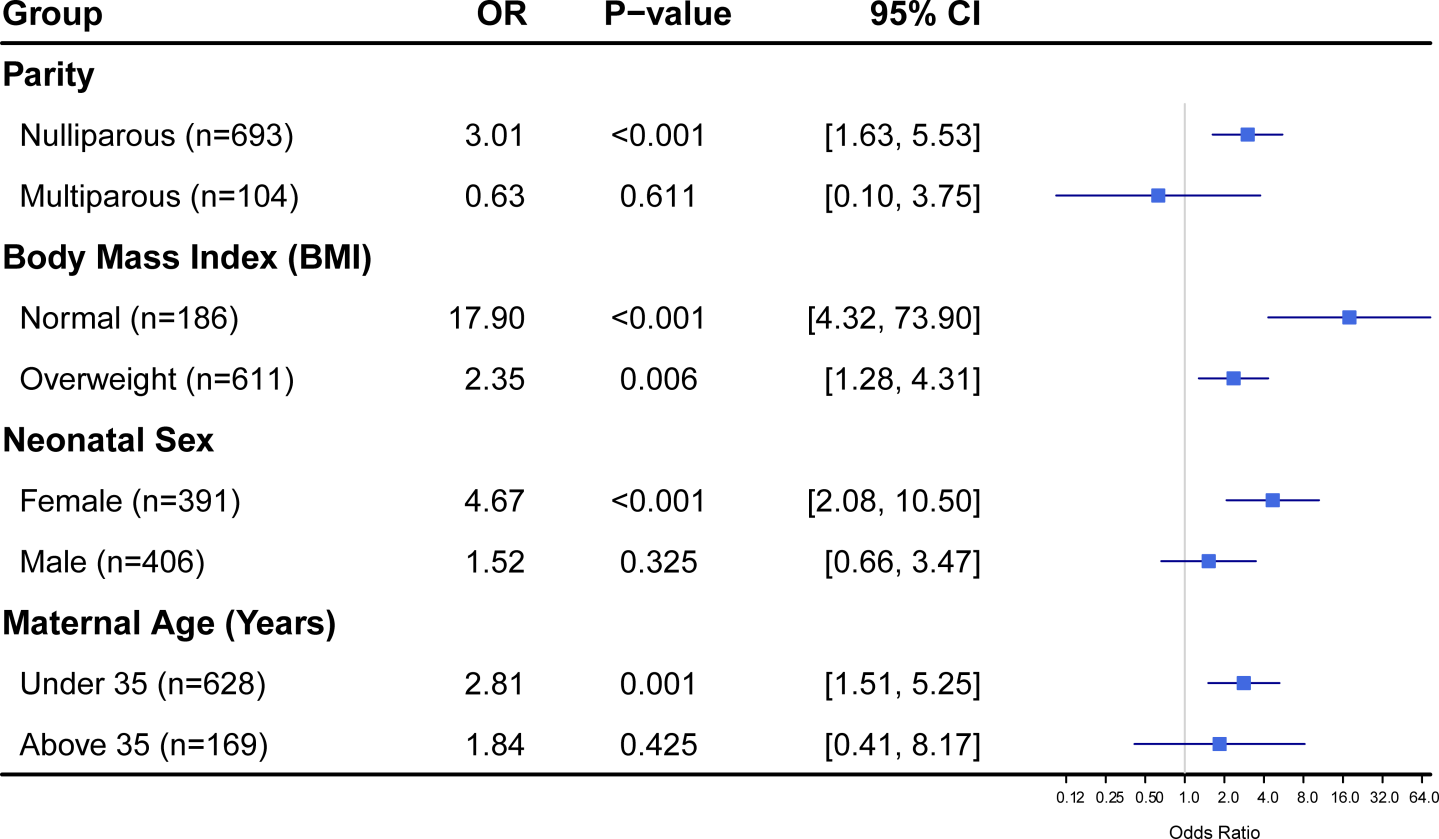
Subgroup analysis of the association between maternal FT3/FT4 ratio and the risk of neonatal adverse outcomes.

To evaluate the robustness of our main findings and identify potential vulnerable populations, we performed subgroup analyses stratified by maternal parity, body mass index (BMI), neonatal sex, and maternal age, as illustrated in **Figure 2**. In terms of maternal reproductive history and age, the adverse impact of an elevated FT3/FT4 ratio appeared to be more prominent in nulliparous women and those under the age of 35. Specifically, among nulliparous mothers (n=697), a higher FT3/FT4 ratio was strongly associated with a threefold increase in the odds of neonatal adverse outcomes (OR = 3.01, 95% CI: 1.63–5.53, *P* < 0.001). Conversely, this association was attenuated and lost statistical significance in multiparous women (OR = 0.63, *P* = 0.611). A similar divergence was observed regarding maternal age; the risk was significantly elevated in the younger cohort (<35 years; OR = 2.81, *P* = 0.001), whereas in mothers aged 35 and above, the relationship was not statistically significant (OR = 1.84, *P* = 0.425), suggesting that younger primiparous women might be more sensitive to thyroid hormone imbalances.

Stratification by metabolic status revealed that the positive association between the FT3/FT4 ratio and neonatal outcomes persisted across BMI categories but exhibited a dramatic difference in magnitude. In the overweight group (n=619), the association was significant and moderate (OR = 2.35, 95% CI: 1.28–4.31, *P* = 0.006). However, in mothers with a normal BMI (n=178), the effect size was strikingly larger (OR = 17.90, *P* < 0.001). It is worth noting that the confidence interval for the normal BMI group was notably wide [4.32, 73.90], likely due to the smaller sample size in this subgroup, yet the lower bound of the interval remained well above unity, reinforcing the strength of the association in non-obese individuals.

Furthermore, the analysis stratified by neonatal sex indicated a potential sex-specific susceptibility to maternal thyroid dysfunction. The association was highly significant among female neonates (n=391), with an odds ratio of 4.67 (95% CI: 2.08–10.50, *P* < 0.001). In contrast, for male neonates (n=406), while the direction of the effect remained positive (OR = 1.52), the association did not reach statistical significance (*P* = 0.325). This disparity suggests that female fetuses might be biologically more vulnerable to the specific thyroid hormone profile characterized by a high FT3/FT4 ratio during gestation.

## 4 Discussion

In this retrospective cohort study, we identified a robust and independent association between an elevated maternal free triiodothyronine-to-free thyroxine (FT3/FT4) ratio and an increased risk of NAOs. Multivariable logistic regression analyses demonstrated that mothers in the highest quartile (Q4) of the FT3/FT4 ratio had over 2.5-fold increased odds of NAOs compared to those in the lowest quartile (Q1), even after adjusting for confounders including maternal BMI, age, gravidity, parity, hematological parameters, and neonatal factors. Our baseline analysis revealed notable differences across FT3/FT4 ratio quartiles that warrant careful interpretation. Women in Q1 were significantly older, had lower body weight, and lower BMI compared to those in higher quartiles. These differences suggest that the FT3/FT4 ratio is intrinsically linked to maternal metabolic status and body composition. Adipose tissue is a metabolically active organ that expresses deiodinases, and variations in maternal adiposity may modulate systemic thyroid hormone metabolism, thereby influencing the FT3/FT4 ratio. Additionally, lower body weight and BMI may reflect reduced nutritional reserves, which could affect the availability of essential cofactors required for optimal deiodinase function (13, 15, 16). This association was particularly pronounced for individual outcomes, with the strongest effect observed for newborn anemia. The pronounced association with neonatal anemia (OR = 4.127, p = 0.003) warrants particular attention. Excessive biologically active FT3 in early pregnancy may exacerbate intrauterine oxidative stress, potentially disrupting fetal erythropoiesis or inducing hemolysis in the neonatal period. Thyroid hormones play a well-documented role in fetal erythropoiesis: FT3 stimulates erythroid progenitor cell proliferation and differentiation through direct action on thyroid hormone receptors expressed in bone marrow and hepatic hematopoietic tissue during fetal development. Excessive maternal FT3 exposure may disrupt this finely regulated process through several potential pathways (17). Furthermore, elevated FT3 levels and altered metabolic states may be linked to increased oxidative stress, potentially leading to shortened erythrocyte lifespan or mild hemolysis, contributing to neonatal anemia (18). It is also plausible that the observed association is partially mediated by other adverse outcomes such as preterm birth or low birth weight, which are themselves strongly associated with neonatal anemia. Maternal thyroid dysfunction may impair placental iron transport by altering the expression of transferrin receptors and hepcidin regulatory pathways, thereby limiting fetal iron availability for hemoglobin synthesis (19). Furthermore, maternal metabolic status, as reflected by BMI and body composition differences across quartiles, may interact with thyroid hormone metabolism to influence fetal erythropoiesis (20). Future studies measuring cord blood thyroid hormone levels, iron status markers, and erythropoietin concentrations would help clarify these mechanistic pathways. This threshold-dependent effect, visualized by our RCS analysis, suggests that beyond a ratio of 0.30, the maternal thyroid profile may shifts from a physiological adaptation to a potential contributor tofetal metabolic dysregulation. RCS analyses further revealed a non-linear “J-shaped” dose-response relationship, with risk escalating sharply beyond a ratio of approximately 0.30, underscoring the threshold-dependent nature of this biomarker. The RCS model’s “J-shaped” pattern indicates that at lower FT3/FT4 ratios (below ~0.25), the risk of NAOs remains stable and minimal, suggesting a physiological range where maternal thyroid hormone conversion likely supports fetal development without excess. However, beyond this threshold, particularly above 0.30, the curve demonstrates a rapid increase in NAO probability, potentially reflecting a shift to pathological overactivation of peripheral deiodinase enzymes, leading to disproportionate FT3 production. This excess biologically active FT3 may interfere with fetal hematopoiesis by potentially inducing oxidative stress, impairing erythrocyte maturation, or promoting hemolysis in the neonatal period, as evidenced by the high OR for anemia, which persisted across models and showed a linear trend in individual RCS analysis (21). Similarly, for cardiovascular outcomes like PDA (OR = 2.408) and myocardial injury (OR = 1.780), elevated FT3 exceeding this threshold could disrupt normal ductal closure or cardiac maturation through altered vascular tone or increased metabolic demand, consistent with observations in preterm infants where thyroid dysfunction delays PDA resolution (22).

Our results align with emerging evidence linking maternal thyroid hormone imbalances to adverse fetal and neonatal outcomes. For instance, a higher FT3/FT4 ratio has been associated with increased risks of gestational diabetes mellitus (GDM) and related complications, such as large-for-gestational-age (LGA) infants and preeclampsia, in euthyroid pregnant women (13). In that study, conducted in a Caucasian cohort, the upper tertile of the ratio at 26–28 weeks gestation correlated with a twofold increase in GDM odds and higher rates of cesarean sections and neonatal intensive care unit (NICU) admissions, mirroring our observations of elevated NAO risks in the highest quartile. Similarly, longitudinal analyses have shown that elevated FT3 and FT3/FT4 ratios early in pregnancy are indicative of metabolic dysregulation, potentially driving insulin resistance and hyperglycemia, which may indirectly contribute to neonatal complications like jaundice and anemia (23). These associations persist postpartum, with persistent adverse metabolic profiles in affected mothers, suggesting a long-term impact on maternal-fetal health.

The observed link between the FT3/FT4 ratio and NAOs may be mediated by enhanced peripheral deiodinase activity, which converts FT4 to the more biologically active FT3 in response to metabolic stress or systemic inflammation. Baseline characteristics in our cohort revealed that mothers with NAOs had higher pre-pregnancy BMI, diastolic blood pressure, white blood cell counts, and thyroid autoantibody titers, alongside elevated FT3/FT4 ratios. This profile suggests a compensatory thyroid response to obesity-related inflammation, as supported by studies demonstrating positive correlations between maternal BMI and both maternal and neonatal FT3/FT4 ratios, which in turn may influence birth weight (24). Furthermore, thyroid autoimmunity, evident in our NAO group through elevated autoantibodies, may exacerbate this imbalance by impairing thyroid function and promoting a pro-inflammatory state, potentially leading to placental dysfunction and restricted fetal growth. This is consistent with reports of maternal hypothyroidism increasing the risk of preterm delivery and low birth weight, even in subclinical forms (25).

Subgroup analyses in our study provided insights into vulnerable populations, revealing stronger associations in nulliparous women under 35 years (OR = 3.01, p < 0.001), those with normal BMI (OR = 17.90, p < 0.001), and female neonates (OR = 4.67, p < 0.001). These findings suggest sex-specific fetal susceptibility, possibly due to differential placental thyroid hormone transport or deiodinase expression, with female fetuses exhibiting greater vulnerability to excess FT3 exposure. This aligns with evidence of sex-dimorphic effects in thyroid-mediated outcomes, where female neonates show heightened risks of congenital hypothyroidism or altered TSH levels in response to maternal thyroid resistance (26). The pronounced effect in normal-BMI women contrasts with the attenuated risk in overweight individuals, potentially indicating that obesity-related adaptations mask the ratio’s impact, as observed in cohorts where higher FT3/FT4 correlates with birth weight in gestational transient thyrotoxicosis (GTT) but only after maternal weight adjustment (27).Interestingly, the magnitude of association was significantly higher in women with a normal BMI compared to those who were overweight (OR 17.90 vs. 2.35). This disparity may stem from the ‘desensitization’ of deiodinase enzymes in the context of chronic obesity-related inflammation. Conversely, in non-obese individuals, the fetal-placental unit may be more acutely sensitive to sudden shifts in maternal thyroid hormone conversion, making the FT3/FT4 ratio a more potent predictor in this subgroup. In that study, a positive correlation between the ratio and birth weight (r=0.317, p=0.017) was noted in GTT cases, suggesting beneficial effects at moderate elevations but adverse outcomes at extremes, as seen in our “J-shaped” curve.

The non-linear relationship identified via RCS reinforces the notion of a threshold effect, where risks remain low below a ratio of 0.25 but surge thereafter. This pattern was heterogeneous across individual NAOs: non-linear for jaundice (P = 0.011) but linear for anemia (P = 0.643), with high model discrimination (C-index up to 0.948). Such dose-response dynamics are echoed in population-based cohorts linking low maternal FT4 and high T3 to large-for-gestational-age risks, implying that imbalances in thyroid hormone conversion might disrupt fetal growth trajectories (28). Additionally, the role of FT3 in glucose metabolism—enhancing hepatic gluconeogenesis and peripheral uptake—may explain associations with jaundice and anemia, as excess FT3 could induce hemolysis or oxidative stress in neonates (29).

Strengths of our study include the comprehensive adjustment for confounders, sensitivity analyses excluding potential mediators, and the use of RCS for nuanced dose-response modeling. The high C-indices (0.818–0.948) indicate robust predictive utility. Despite the findings, this study has limitations. First, the observational design precludes definitive causal inference. Second, the single-center retrospective design inherently introduces potential selection bias and limits the external validity of our findings. Our cohort was drawn exclusively from a tertiary care center in China, where regional iodine nutrition status and local clinical practices may differ from other regions. Our results may not be generalizable to populations with different ethnic backgrounds, healthcare systems, or nutritional status. Additionally, while our multivariable models adjusted for numerous established confounders, residual confounding from unmeasured variables—such as maternal dietary patterns, environmental exposures, and genetic polymorphisms affecting deiodinase activity—cannot be entirely excluded. Finally, the retrospective nature of our study precludes causal inference; while we identified robust associations, we cannot establish that altered thyroid hormone metabolism directly causes these adverse events. Additionally, the cohort was predominantly from a single center in China, limiting generalizability to diverse ethnic groups; future multi-ethnic studies are warranted. Furthermore, our exclusion of women receiving levothyroxine or antithyroid medications, while necessary to isolate physiological thyroid hormone variations, limits the generalizability of our findings to the broader obstetric population. In real-world practice, levothyroxine supplementation primarily increases circulating FT4 levels, which inherently lowers the FT3/FT4 ratio. This pharmacological effect may fundamentally alter the relationship between the FT3/FT4 ratio and neonatal outcomes compared to the physiological state observed in our untreated cohort, as exogenous supplementation may affect peripheral deiodinase activity and tissue-level sensitivity differently than endogenous adaptations. Future prospective studies should investigate whether our findings extend to treated populations with subclinical or overt thyroid dysfunction.

## Data Availability

The raw data supporting the conclusions of this article will be made available by the authors, without undue reservation.
