# Supplementary material for: The association between maternal FT3/FT4 ratio in early pregnancy and adverse neonatal outcomes: a retrospective cohort study
Source: Front Endocrinol (Lausanne). 2026 May 12;17:1802981. doi: 10.3389/fendo.2026.1802981 (PMC13201191; doi:10.3389/fendo.2026.1802981)
Supplement: Supplementary file 1 [file DataSheet1.pdf]

Supplementary Table S1. Detailed Exclusion Criteria

Category	Specific Conditions Excluded
**Thyroid History**	Hypothyroidism, hyperthyroidism, thyroiditis, prior thyroid surgery.
**Autoimmune/Systemic**	Systemic lupus erythematosus (SLE), rheumatoid arthritis, antiphospholipid syndrome, Sjögren’s syndrome, known autoimmune thyroid disease.
**Preexisting Comorbidities**	Pregestational diabetes mellitus, chronic hypertension, chronic kidney disease, chronic liver disease, cardiovascular disease.
**Obstetric Factors**	Multiple gestations, assisted reproductive technology (ART) conception.
**Medication Use**	Antithyroid drugs, levothyroxine, amiodarone, glucocorticoids, immunosuppressants.
**Other**	Iodine supplementation at pharmacologic doses, fetal congenital anomalies (diagnosed prenatally or at birth).

Supplementary Table S2. Components of the Composite Neonatal Adverse Outcome (NAO) Endpoint and Event Frequencies

(Note: Infants may have multiple diagnoses; thus, the sum of events exceeds the total number of affected infants.)

Diagnostic Category	Specific Diagnosis	No. of Cases (n=797)
**Neurologic**	Hypoxic–ischemic encephalopathy	140
	Intraventricular hemorrhage	5
	Subarachnoid/Subdural/Intracranial hemorrhage	14
**Respiratory**	Acute respiratory distress syndrome	110
	Respiratory failure	64
	Meconium aspiration syndrome	9
	Pulmonary hemorrhage	12
	Bronchopulmonary dysplasia	2
	Persistent pulmonary hypertension	6
**Cardiovascular**	Myocardial injury	233
	Patent ductus arteriosus	80
	Heart failure	1
**Infectious**	Idiopathic perinatal infection	85
	Neonatal sepsis	20
**Hematologic/Metabolic**	Neonatal hypoglycemia	100
	Anemia of prematurity	85
	Neonatal anemia	40
	Polycythemia	2
**Gastrointestinal**	Gastrointestinal hemorrhage	10
	Necrotizing enterocolitis	1
**Other**	Perinatal death	3
	Congenital anomalies*	20

Supplementary Table S3. Baseline Characteristics of Participants According to Neonatal Adverse Outcomes

Variable	Without Neonatal Adverse Outcome (n = 419)	Neonatal Adverse Outcome (n = 378)	p-value
Maternal age (years)[a]	30.43 (3.67)	30.62 (3.93)	0.472
Height (cm)[a]	163.30 (5.70)	163.16 (4.89)	0.707
Weight (kg)[a]	73.91 (10.53)	75.62 (12.39)	0.036
Body Mass Index (BMI)[a]	27.75 (4.05)	28.36 (4.32)	0.040
Systolic blood pressure (mmHg)[b]	110.00 [110.00, 120.00]	117.00 [110.00, 119.75]	0.060
Diastolic blood pressure (mmHg)[b]	70.00 [70.00, 80.00]	78.00 [70.00, 80.00]	0.013
Gravidity (G)[a]	1.60 (0.86)	1.75 (0.97)	0.025
Parity (P)[a]	1.13 (0.37)	1.14 (0.38)	0.844
White blood cell count ($\times 10^9$/L)[a]	9.68 (2.82)	11.01 (3.18)	<0.001
Platelet count ($\times 10^9$/L)[a]	201.88 (51.60)	205.22 (59.84)	0.398
Serum creatinine (μmol/L)[b]	45.90 [41.00, 51.95]	47.00 [40.80, 52.90]	0.349
Uric acid (μmol/L)[b]	281.45 [236.28, 323.88]	275.80 [234.00, 342.20]	0.351
Free triiodothyronine (FT3, pmol/L)[b]	4.23 [3.91, 4.59]	4.64 [4.25, 4.98]	<0.001
Free thyroxine (FT4, pmol/L)[b]	14.51 [13.01, 16.04]	15.04 [13.77, 16.46]	<0.001
FT3/FT4 ratio[b]	0.29 [0.27, 0.32]	0.31 [0.28, 0.34]	<0.001
Thyroid-stimulating hormone (TSH, mIU/L)[b]	1.63 [1.07, 2.30]	1.46 [0.97, 2.13]	0.017
Thyroglobulin antibody (TgAb, IU/mL)[b]	0.90 [0.90, 12.01]	16.48 [10.61, 27.83]	<0.001
Anti-thyroid peroxidase antibody (TPOAb, IU/mL)[b]	0.50 [0.25, 28.00]	28.00 [10.37, 35.40]	<0.001
Neonatal birth weight (g)			
≥2500	404 (98.1)	226 (60.4)	<0.001
1500–2499	8 (1.9)	124 (33.2)	
1000–1499	0 (0.0)	20 (5.3)	
<1000	0 (0.0)	4 (1.1)	

Gestational age at delivery (weeks)			
≥37	399 (97.3)	180 (49.2)	<0.001
34–36	8 (2.0)	120 (32.8)	
32–34	2 (0.5)	34 (9.3)	
<32	1 (0.2)	30 (8.2)	
<28	0 (0.0)	2 (0.5)	
Neonatal sex			
Male	201 (48.0)	205 (54.2)	0.090
Female	218 (52.0)	173 (45.8)	
Maternal outcome			
No complications	258 (61.6)	88 (23.3)	<0.001
Complications	161 (38.4)	290 (76.7)	

Variables are presented as mean ± SD “a”or median [IQR] “b”. Statistical significance was set at $p < 0.05$.

Supplementary Table S4. Sensitivity analysis of the association between maternal FT3/FT4 ratio quartiles and neonatal outcomes, excluding potential mediators.

FT3/FT4 Quartile	OR (95% CI)	P-value
Q1 (Reference)	1.00 (Reference)	-
Q2	1.210 (0.789 – 1.854)	0.382
Q3	1.200 (0.785 – 1.835)	0.400
Q4	2.218 (1.431 – 3.438)	< 0.0001

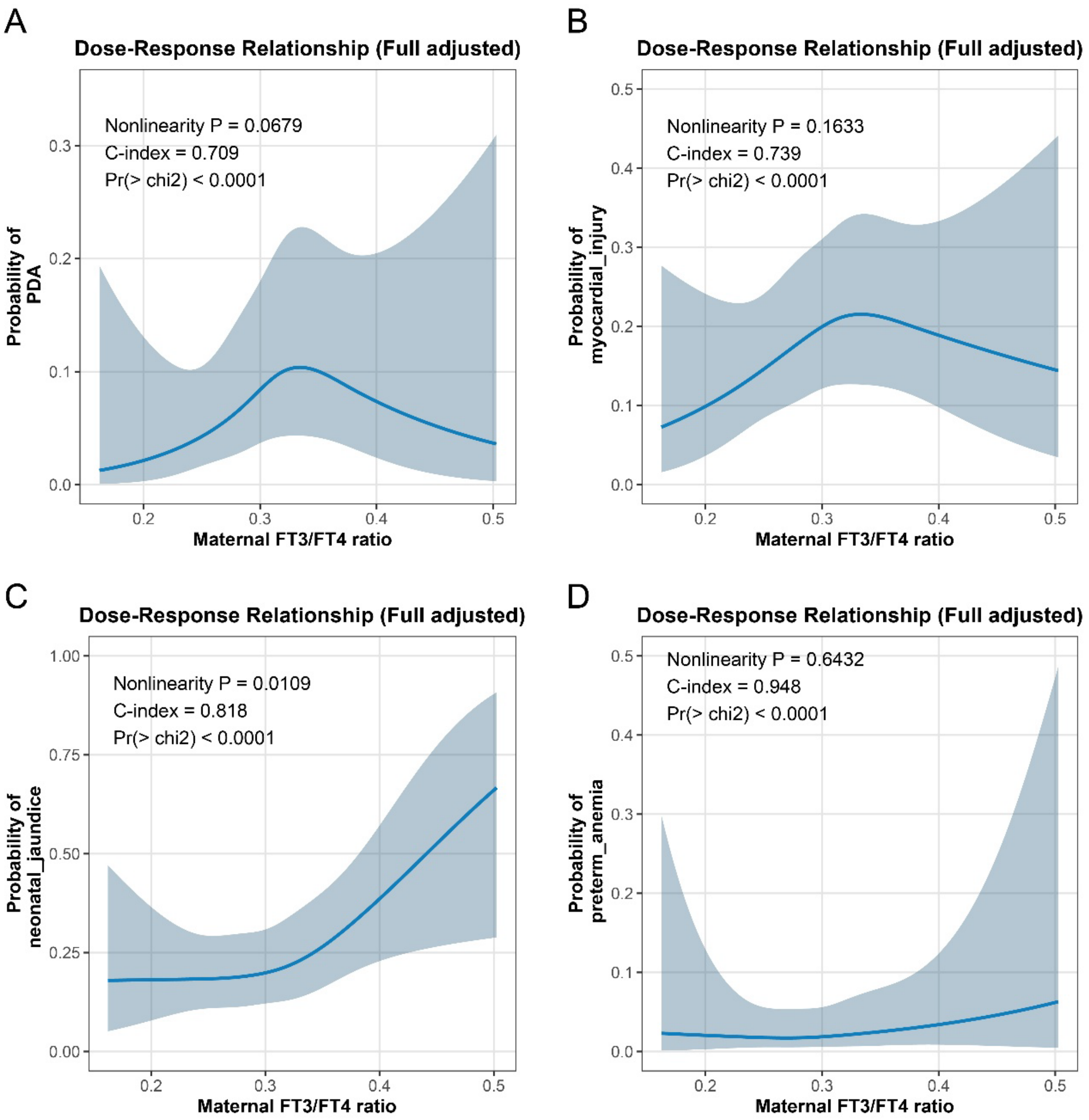

Supplementary Figure S1. Dose-response relationships between maternal FT3/FT4 ratio and specific neonatal adverse outcomes. Restricted cubic spline analyses illustrating the predicted probability of **(A)** patent ductus arteriosus (PDA), **(B)** myocardial injury, **(C)** neonatal jaundice, and **(D)** preterm anemia across the distribution of maternal FT3/FT4 ratios.
